# Doctors and their families

**DOI:** 10.1177/10398562231151849

**Published:** 2023-01-18

**Authors:** Susan Mary Benbow

**Affiliations:** Centre for Ageing and Mental Health, 11965 University of Chester, Chester, UK; Older Mind Matters Ltd, Manchester, UK

**Keywords:** coordinated management of meaning, family, resilience, well-being

## Abstract

**Objective:**

This article reflects on the relationship between doctors and their families and how it influences a doctor’s health, well-being and practice and the health and well-being of other family members. It uses an established model for conceptualising this recursive relationship, drawing on systemic and communications theory, coordinated management of meaning. The article invites doctors to reflect on relational influences between them and their families across the course of their career and following retirement.

**Conclusion:**

Families are important to, and influence, the well-being of their doctor-members. Likewise, doctors are important to, and influence, the health and well-being of their families.

Medicine, as a career, requires a high investment of mental effort and time. As a result, many doctors strongly identify with their profession and this professional identification often endures, even following retirement from medical practice.^1^

One of the core activities of a practising doctor is prescribing medicines. In the United Kingdom doctors are required by the General Medical Council to avoid prescribing for themselves and for family (‘anyone you have a close personal relationship with’^2^ page 9): this is equally the case in Australia^3^ and New Zealand,^4^ but, despite this, the very fact of being a doctor or having a doctor in the family may influence family members’ well-being practices, health seeking and use of services.

Doctors live and work within the broader context of their families, whether living with family members or not – even if family is absent for some reason (such as death or estrangement), that absence and history is part of the context. Becoming a doctor brings with it knowledge and responsibilities. Family members are aware of that knowledge and may seek it out^5^ (or deliberately make the choice not to seek it out and to go against what they would expect the doctor-family-member to say or think). In seeking (or not seeking) knowledge out, they inevitably place responsibilities on the doctor-family-member and their relationship with that individual. Thus, a doctor-family-member influences their family, which might consist of several generations, and the family influences the doctor-family-member, in terms of health, beliefs about health and health systems, and also possibly individuals’ well-being practices.

This bidirectional influence can have both positive and negative effects. For example, younger family members may rebel against well-being messages and choose life-style behaviours that doctor-family-members regard as threatening to health, for example, smoking and misuse of substances. This may signify to a doctor-parent or doctor-sibling that they have failed, not only as a parent/sibling but as a member of the medical profession, with implications for that doctor’s well-being, sense of self, professional identity and medical practice.

Furthermore, despite the guidance from the General Medical Council in the United Kingdom ^2^ and other regulatory agencies worldwide,^3,4^ doctors are notorious for treating themselves ^6^ and their own families. A number of potential factors may operate here: illness and the associated need for treatment may carry stigma or may be regarded as a sign of ‘weakness’ or failure, and professional loyalty and peer pressure may influence decisions about consulting a colleague regarding treatment of self or family members.^7,8^ There are many reasons why a doctor treating their family members is likely to be unwise, including for the doctor, lack of professional objectivity, feeling obligated to provide care even if not qualified to do so. For the family member there are issues of patient autonomy, informed consent and continuity of care.^9^

Conversely, there are positive effects of having a doctor in the family. Walsh defines family resilience as *the capacity to withstand and rebound from disruptive life challenges*,^10^ and this would include illness in family members. A doctor-family-member may contribute to family resilience in a range of ways. For example, the doctor may encourage (and perhaps model) compliance with well-being practices such as vaccinations and preventive medications, and encourage family members to seek information and understanding about their health conditions and, in some situations, may advocate for them. Doctors in the family may assist in mobilising appropriate family (and other) support for sick family members at times of health challenges and in withdrawing that help and encouraging independence when a family member recovers. They may be more able to speak with family members about death and dying so that family members can make end-of-life choices for themselves. With regards to positive effects on family health, a study in Sweden^11^ suggests a strong relationship between exposure to health-related expertise (in terms of having an individual with a health professional degree, physician or nurse, in the family) and family health. The findings included that, amongst older relatives, life-style related diseases were found to be less common and adherence to preventive medications was greater, and that amongst younger relatives there was a greater likelihood of preventive health investments.

At the same time the family and close relationships contribute to the doctor’s work-life balance, their health and well-being,^12^ although many doctors struggle with achieving a work-life balance and with their ability to manage family and caring commitments as illustrated by a recent Irish study.^13^

## A model for conceptualising doctors’ impact within families

**Figure 1.**
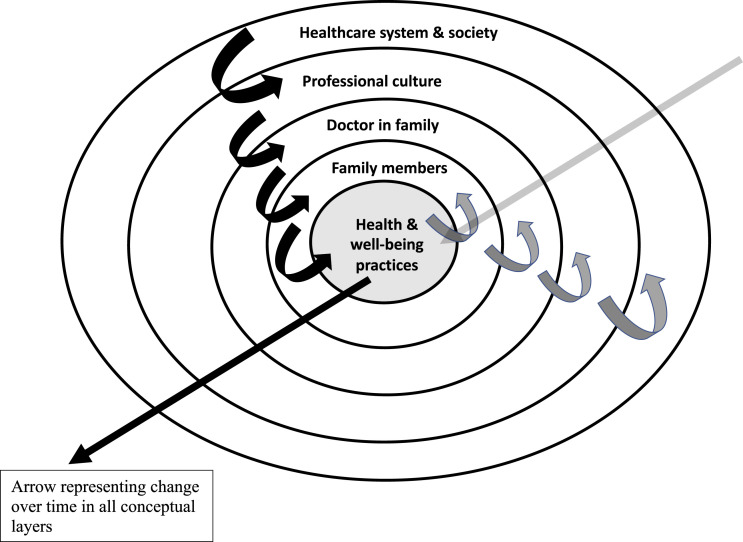
Some of the different contexts influencing health and well-being practices in a family (modified from coordinated management of meaning^14^). *Note:* Black curved arrows indicate contextual forces Grey curved arrows indicate implicative forces.

Useful models for understanding the effect of having a doctor in the family can be found in both systems theory and communications theory. Coordinated management of meaning is a way of exploring the various influences on meanings, and how meanings are constructed and communicated.^14^ Applied here it suggests that a doctor within a family will influence family relationships, episodes of ill-health and well-being practices by their communications and behaviours which draw on the doctor’s training, knowledge and experience. Figure 1 represents how the model can be applied. It puts health and well-being practices at the centre and black arrows indicate how contextual forces are exerted from broader/higher contexts towards a family member’s health and well-being practices. The grey arrows indicate that there are also implicative forces (traditionally regarded as weaker) in the opposite direction. Thus, the model also recognises that the communications and behaviours of family members will likewise influence the doctor: for example, a doctor-parent may believe that they have a close relationship with their teenaged son, but, when they discover that he has regularly used substances without their knowledge, they redefine the parent-son relationship and might regard themselves differently as a parent. In addition, the doctor’s beliefs and approach to people who have problems with substance use and their family members may well change too.

In family and systemic therapy, coordinated management of meaning offers a helpful way of putting meanings in relationships into broader contexts.^15,16^ In families with a doctor-member the relationship with the doctor-member will influence other family members, and in turn the relationship with other family members will influence the doctor-family-member.

A limitation of Figure 1 is that the model is represented two-dimensionally and does not easily incorporate change over time. The straight arrows on the figure signify movement and change with the passage of time. As a doctor progresses from medical student through their working life to the post-retirement phase of their career, and the family moves through the family life cycle,^17^ there will be changes, not only in the family, but in professional culture, the healthcare system and society.

## Anonymised illustrative examples

Case 1 A member of an extended medical family had an acute episode of mental illness and was admitted to a psychiatric unit. Doctor-members of the family were involved in persuading the ill family member to accept admission and treatment. They experienced what it was like to be concerned relatives and carers and the sense of exclusion and being discounted that carers had described to them in their work contexts. The family member’s illness and attitudes and beliefs about it also influenced the doctor-members of the family and are likely to have impacted on their future work.

Case 2 A member of an extended family with a family member working in psychiatry took their own life in the context of an unrecognised severe depressive illness. This confronted the psychiatrist family member with the shocking reality that our own families are not immune from the illnesses and tragedies we work with, along with a profound sadness that, despite their knowledge, training and expertise, they had not been aware of what was happening and had failed to intervene in what they suspected had been a potentially treatable illness.

These examples relate to mental ill-health but could equally have involved physical ill-health.

## Conclusions

Doctors’ self-prescribing and prescribing for friends and family is universally regarded as poor practice and to be avoided. Families contribute to our resilience, health and well-being as doctors and simultaneously challenge us. Harrist and colleagues^18^ write that family resilience occurs when … *family systems engage in processes that foster positive adaptation in the overall family system, family subsystem, (and) individual family members…* Doctors contribute to the resilience of their families whilst challenging it, and families contribute likewise to their doctor-family-member. We underestimate the importance of our families to our health and well-being, and our potential influence on them, at our peril.
